# Enhanced microbubble contrast agent oscillation following 250 kHz insonation

**DOI:** 10.1038/s41598-018-34494-5

**Published:** 2018-11-05

**Authors:** Tali Ilovitsh, Asaf Ilovitsh, Josquin Foiret, Charles F. Caskey, Jiro Kusunose, Brett Z. Fite, Hua Zhang, Lisa M. Mahakian, Sarah Tam, Kim Butts-Pauly, Shengping Qin, Katherine W. Ferrara

**Affiliations:** 1 Department of Radiology, Stanford University, Palo Alto, California USA; 2 Department of Biomedical Engineering, University of California, Davis, California USA; 3 Vanderbilt University Institute of Imaging Science, Nashville, Tennessee USA; 4 Department of Radiology and Radiological Sciences, Vanderbilt University Medical Center, Nashville, Tennessee USA

## Abstract

Microbubble contrast agents are widely used in ultrasound imaging and therapy, typically with transmission center frequencies in the MHz range. Currently, an ultrasound center frequency near 250 kHz is proposed for clinical trials in which ultrasound combined with microbubble contrast agents is applied to open the blood brain barrier, since at this low frequency focusing through the human skull to a predetermined location can be performed with reduced distortion and attenuation compared to higher frequencies. However, the microbubble vibrational response has not yet been carefully evaluated at this low frequency (an order of magnitude below the resonance frequency of these contrast agents). In the past, it was assumed that encapsulated microbubble expansion is maximized near the resonance frequency and monotonically decreases with decreasing frequency. Our results indicated that microbubble expansion was enhanced for 250 kHz transmission as compared with the 1 MHz center frequency. Following 250 kHz insonation, microbubble expansion increased nonlinearly with increasing ultrasonic pressure, and was accurately predicted by either the modified Rayleigh–Plesset equation for a clean bubble or the Marmottant model of a lipid-shelled microbubble. The expansion ratio reached 30-fold with 250 kHz at a peak negative pressure of 400 kPa, as compared to a measured expansion ratio of 1.6 fold for 1 MHz transmission at a similar peak negative pressure. Further, the range of peak negative pressure yielding stable cavitation *in vitro* was narrow (~100 kPa) for the 250 kHz transmission frequency. Blood brain barrier opening using *in vivo* transcranial ultrasound in mice followed the same trend as the *in vitro* experiments, and the pressure range for safe and effective treatment was 75–150 kPa. For pressures above 150 kPa, inertial cavitation and hemorrhage occurred. Therefore, we conclude that (1) at this low frequency, and for the large oscillations, lipid-shelled microbubbles can be approximately modeled as clean gas microbubbles and (2) the development of safe and successful protocols for therapeutic delivery to the brain utilizing 250 kHz or a similar center frequency requires consideration of the narrow pressure window between stable and inertial cavitation.

## Introduction

Microbubbles (MBs) composed of a gas core with a stabilizing shell serve as contrast agents for ultrasound imaging. Each MB is a few microns in diameter, and hence can be safely injected intravenously. At frequencies that match the range of clinical ultrasound, MBs are highly echogenic due to the impedance mismatch between the MB and the surrounding tissue and their near resonant oscillation. This oscillation yields a strong returned echo from an incident ultrasound pulse, with single MBs detected within vessels *in vivo*. Therefore, MBs are extensively used for ultrasound imaging^1^, and more recently, for super resolution imaging^2,3^. In addition to imaging, MBs are also used for therapeutic applications including drug delivery^4^, gene-based therapy^5,6^, and blood brain barrier (BBB) opening^7–9^. Upon ultrasound excitation, MBs cavitate and pulsate volumetrically. Among other parameters, the oscillation dynamics depend on the acoustic peak negative pressure (PNP), frequency, and MB diameter. Optical observations of MBs have been used to study their radial oscillations^10^, translation^11^, and fragmentation^12–15^ for both free and adherent MBs^16,17^.

The safety of MB-based treatments is assessed by monitoring MB cavitation. MBs under stable and inertial cavitation have distinct acoustical signatures that are detected by real-time feedback provided by MBs echoes^9,18,19^. At a low acoustic pressure, the MB gas is compressed and expanded repeatedly, a process known as stable cavitation. During long-lasting stable cavitation oscillations, adjacent cell membranes are temporarily altered, facilitating therapeutic delivery^20^. This process is associated with microstreaming surrounding the MBs that can reversibly modify the permeability of the cell membranes^21^. At a high acoustic pressure, inertial cavitation occurs. The MBs rapidly collapse and will be destroyed via fragmentation into smaller MBs or gas diffusion. MB collapse can produce liquid jets that release high levels of energy and may cause severe mechanical damage to the surrounding tissues because of its associated high internal temperature, pressure, and velocity jets^22–25^. Inertial cavitation may result in thermal and/or mechanical injury to normal brain adjacent to the clinical target and may result in devastating and potentially fatal intracranial hemorrhage.

MB excitation is typically performed in the frequency range of commercial ultrasound imaging (1–5 MHz). A lower transmission center frequency is desirable to enhance the penetration depth and enlarge the focal zone. Clinical therapeutic ultrasound systems typically operate with a center frequency between 650 kHz and 1.5 MHz^26–28^. Yet, systems using a center frequency of ~220 kHz are now entering clinical and human use^29,30^. For brain therapy applications, the lower frequency aids in focusing through the human skull to a predetermined location with minimal distortion and attenuation, as compared to higher frequencies^31^ and eliminates the need for extensive patient-specific correction^32^. In addition, for the undersampled transducer arrays used in therapeutic ultrasound, the use of a lower frequency also facilitates beam steering by reducing grating lobes. However, the oscillation of MB contrast agents has not been fully assessed in this frequency range.

The mechanical index^33^ (PNP divided by the square root of the center frequency) and the cavitation index^34^ (PNP divided by the center frequency) are parameters that were derived to gauge the likelihood of adverse bioeffects from inertial cavitation and monitor the MB activity during stable cavitation. The inverse dependence of these parameters on the ultrasound center frequency predicts larger adverse bioeffects at lower frequencies. The resonant size of typical MBs is at frequencies between 2 and 10 MHz^35^. Therefore, for frequencies lower than 800 kHz, the MBs are significantly smaller than the resonance wavelength. In the past it was assumed that below 800 kHz, the mechanical and cavitation indices will not be valid, and that larger acoustic pressures will be required to initiate stable and inertial cavitation^34,36,37^. However, for a free gas bubble, the response to quasistatic pressure changes in the liquid (i.e. excitation well below the natural resonance frequency) shows that a rarefactional pressure threshold exists above which unstable equilibrium conditions arise. The pressure at which this growth occurs is known as the Blake threshold and is a consequence of the fact that an unshelled MB in liquid cannot withstand arbitrarily high static tension induced by rarefactional pressure^38–40^. *In vivo* BBB opening studies with a center frequency of ~250 kHz in rabbits^7,41^ and macaques^42^, suggest that the window in which increased BBB permeability can be safely achieved is narrow, and report tissue damage above a relatively low PNP of 300 kPa. Therefore, in order to use this low frequency safely, there is a need to characterize the oscillation of shelled MBs following transmission of an ultrasound pulse with a center frequency near 250 kHz.

Here, characterization of MB dynamics was performed through theoretical predictions, ultra high-speed optical imaging and passive cavitation detection of single oscillating MBs. Specifically, MB resting radii of ~0.75 and ~1.5 µm, were selected and investigated to approximate the radii of Definity and Optison (each commercially available) MBs. Finally, *in vivo* blood brain barrier opening was studied with transcranial ultrasound in mice.

## Materials and Methods

Modeling of the MB oscillation at 250 kHz is of primary importance in predicting the stable cavitation range for treatment. The basis of MB oscillation models is the Rayleigh-Plesset Equation (RPE) for a clean (unencapsulated) MB^43,44^. Previous experimental results^13,23,45^ supported the validity of the RPE model for insonation at 1 MHz. More elaborate models incorporate nonlinearities in shell elasticity and viscosity^46^. Here, the numerical evaluations were performed using two models. The first is the modified RPE that estimates the MB radial oscillations while taking into account the effects of liquid compressibility, the shell and the surrounding tissue, as described previously^47^. The second is the Marmottant model, which accounts for buckling and rupture of the shell during oscillation^48^.

To provide a basis of comparison, we designate a lower limit on MB expansion ratio of 1.1 as a value where stable cavitation is established and that generates harmonic echoes (although other lower limits can also be considered). To further facilitate comparison, an expansion ratio upper limit of 3.5 was chosen as representing the regime resulting in crossover between stable and inertial cavitation (previous predictions ranged from 2.3 to 3.5^13,49^).

### Numerical evaluation parameters

At 250 kHz, a preliminary evaluation of the RPE indicates that the expansion ratio can exceed 35-fold at a PNP of 500 kPa. Since the surface area is proportional to the square value of the expansion ratio, at these large expansions, the resulting gas/water interface is exposed and the MBs can be modeled as clean gas (unshelled) MBs using the RPE^46^. In comparison, at 1 MHz, the oscillations at the same PNP range reach a maximal expansion ratio of 4, hence the shell effect is not negligible at this frequency.

All numerical evaluations were performed in MATLAB (Mathworks, Natick, MA). In the modified RPE^47^, a clean gas MB oscillating at a center frequency of 250 kHz was simulated by setting to zero the shell density, shear modulus, viscosity and thickness, as well as the surface tension of the inner radius. The surface tension of the MB outer radius set to that of saline (0.07 N/m). Using the same model, MB oscillations at a center frequency of 1 MHz were simulated according to previously described parameters^47^. Shell density was 1000 kg/m^3^, shell shear modulus was 122 MPa, shell viscosity was 2.5 Pa·s, shell thickness was 1.5 nm, surface tension of the inner radius was 0.04 N/m, and surface tension of the outer radius was 0.056 N/m.

In the Marmottant model, MBs were simulated according to^48^. The shell surface dilatational viscosity was 7.2 × 10^9^ N, the elastic compression modulus was 0.55 N/m, and the initial surface tension of the MB outer radius was set to that of saline (0.07 N/m).

### Experimental design considerations

In this analysis we focus on insonation using 250 kHz which is far below (more than a factor of 10) the resonance frequency (f_c_) for both clean gas and lipid-shelled MBs (Table 1)^50^. For 250 kHz insonation, the onset of high amplitude oscillation is therefore expected to be predicted by the Blake threshold at least for clean gas MBs, and we validate the applicability to lipid-shelled MBs here. Alternatively, a frequency of 1 MHz is less than a factor of ten from the resonance frequency for lipid-shelled MBs, thus the Blake threshold is not applicable for this frequency. The 1 MHz center frequency (closer to resonance) is therefore used as a basis for comparison to 250 kHz excitation (far from resonance).

**Table 1:** The resonance frequency in MHz for clean gas MB and lipid-shelled MB, for resting radii (r_0_) of 0.75 and 1.5 µm.

	r_0_ = 0.75 µm	r_0 _ = 1.5 µm
Clean gas MB	6 MHz	2.5 MHz
Lipid-shelled MB	7 MHz	4.5 MHz

We also consider the Blake threshold for the MB parameters and MBs studied here. The Blake pressure threshold for a clean gas MB in water at room temperature^39^ is given by

1 $${{\rm{P}}}_{{\rm{B}}}={{\rm{P}}}_{{\rm{stat}}}-{{\rm{P}}}_{{\rm{v}}}+\frac{4{\rm{\sigma }}}{3\surd 3{{\rm{r}}}_{0}}{[1+\frac{{{\rm{r}}}_{0}}{2{\rm{\sigma }}}({{\rm{P}}}_{{\rm{stat}}}-{{\rm{P}}}_{{\rm{v}}})]}^{-0.5}$$

where P_B_ is the Blake pressure threshold, r_0_ is the resting radius of the MB, σ is the surface tension, P_stat_ is the static pressure, and P_v_ is the vapor pressure. Here, we set σ to 0.07 N/m, P_stat_ to 100 kPa, P_v_ to 2.33 kPa.

### Microbubble preparation

MBs composed of a gas core containing perfluorobutane (C_4_F_10_) encapsulated in a phospholipid shell, were prepared as reported in^51^ and briefly summarized here. The lipid shell is a combination of disteroylphosphatidylcholine (DSPC), and 1,2-distearoyl-sn-glycero-3-phosphoethanolamine-N-[methoxy(polyethylene glycol)-2000] (ammonium salt) (DSPE-PEG2K) (Avanti Polar Lipids, Alabaster, AL) with a DSPC:DSPE-PEG2K ratio of 90:10 mol/mol. First, MB precursor solution was made using a thin-film hydration method and then was kept in 1 ml vials at 4 °C until use. Upon use, a single MB vial was activated by 45 seconds of shaking using a VIALMIX shaker (Lantheus Medical Imaging). Size selection was performed to separate the larger MBs (radii of above 1 µm), from the smaller MBs (radii of below 1 µm). The separation was performed by centrifuging the suspension at 16 relative centrifugal force for 1 minute, followed by 45 relative centrifugal force for 1 minute. After centrifuging, the turbid liquid at the bottom of the syringe contained the smaller MBs, whereas the MB cake contained the larger MBs. The smaller MB suspension underwent a second purification to remove MBs smaller than 0.5 µm in radii, by centrifuging at 300 relative centrifugal force for 3 minutes, 3 times, and collecting the MB cake. The size and concentration of the purified MBs were measured with a particle counter system (Accusizer 770 A, Particle Sizing Systems, Port Richey, FL). The MBs were used within three hours of their preparation. To confirm the MB stability over this period, the size and concentration was measured with the particle counter system immediately after preparation and at the end of the experiments. The size distribution and concentration changed by less than 5% between the measurements.

For the ultra high-speed imaging experiments, both larger (1.5 µm in radius), and smaller (0.75 µm in radius) MBs were independently studied. For the acoustical measurements and the *in vivo* experiments, the smaller sized MBs were used in order to narrow the distribution.

### Ultra high-speed optical imaging setup

**Figure 1:**
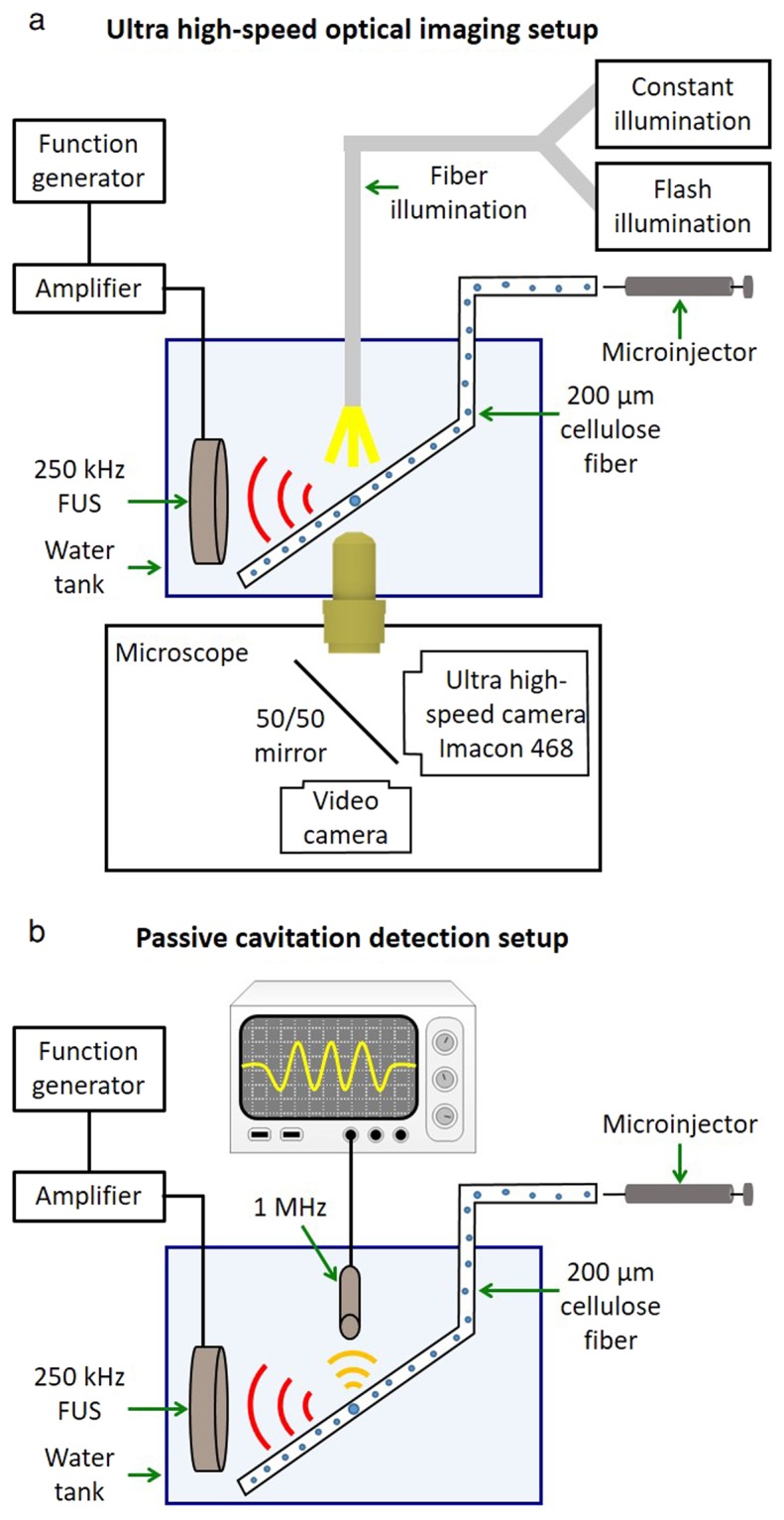
Experimental setups. (**a**) Ultra high-speed optical imaging setup. (**b**) Passive cavitation detection setup.

The experimental setup (illustrated in Fig. 1a) was composed of an inverted microscope (IX70, Olympus, Melville, NY), with a 100x objective and numerical aperture of 1 (Achroplan 100 × , Zeiss, Thornwood, NY). The in-house made lipid-shelled MBs were diluted in saline to approximately 5 × 10^4^ MBs/µl. The diluted solution was pushed through a 200-μm cellulose tube with a manual microinjector (Narishige, Inc., East Meadow, NY). The cellulose tube was placed in a degassed water tank and positioned in the optical field of view of the microscope. The imaged MBs were located at the center of the cellulose fiber. Two transducers were used for insonation. First, a 250 kHz spherically-focused single-element transducer (H115, Sonic Concepts, Bothell, Washington) was placed in the degassed water tank, and aligned to focus on the cellulose fiber in the microscope’s field of view. This transducer has an aperture diameter of 64 mm, a focal distance of 51 mm, f-number of 0.79, lateral full width half maximum (FWHM) of 7 mm and axial FWHM of 50 mm.

The transducer’s pressure was calibrated with a wideband needle hydrophone (HNP-0400, Onda, Sunnyvale, CA, USA; 15% uncertainty in the hydrophone calibration at 250 kHz), and the received pressure signals were displayed on a digital oscilloscope (DPO4034, Tektronix, OR, USA). Here, the pressure corresponding to each predicted expansion was increased slightly (within the error of the hydrophone calibration, 30 kPa) to facilitate comparison between the theoretical and experimental results. Alternatively, the 250 kHz transducer was replaced by a 1 MHz spherically-focused transducer (ILO108HP, Valpey Fisher). This transducer has an aperture diameter of 25.4 mm, a focal distance of 50.8 mm, f-number of 2, lateral FWHM of 3.8 mm and axial FWHM of 46.8 mm. An arbitrary waveform generator (AWG 2021, Tektronix, Wilsonville, OR) was used to generate the desired radio frequency (RF) signal consisting of three to five-cycles of a sinusoid with a 250 kHz or 1 MHz center frequency. The signal was amplified with an RF power amplifier (325LA, ENI, Rochester, NY). Optical images were captured with an ultra high-speed camera (Imacon 468, DRS Hadland, Santa Cruz, CA). The high-speed camera triggered the arbitrary waveform generator, and after a delay equal to the sound propagation time from the transducer to the cellulose fiber, light from a xenon flash illuminated the microscope’s field of view via a 1 mm fiber-optic cable. The ultra high-speed camera captured a streak image (temporal resolution of 28 ns and spatial resolution of 120 nm) of the diameter of the MB as a function of time. Post processing of the images was performed in Matlab to yield the resting MB radius and expansion ratio.

### Passive cavitation detection setup

The spectra of the oscillating MBs echoes were recorded passively using a flat 1 MHz single element transducer (IMO102HP, Valpey Fisher) during interaction with a 250 kHz pulse. Four independent observations were made at each transmitted pressure for each of the frequencies of 250 kHz and 1 MHz. The transducer was positioned perpendicular to the 250 kHz transducer in the water tank and aligned azimuthally to focus at the cellulose fiber, as illustrated in Fig. 1b. The manual microinjector was used to introduce the MBs into the cellulose, at the same dilution of 5 × 10^4^ MBs/µl used in the high-speed camera imaging experiments. The receive echoes were displayed using a digital oscilloscope (DPO4034, Tektronix, OR, USA), and saved for post processing in Matlab. Since therapeutic ultrasound typically uses long waveforms, the transmitted pulse consisted of a 100-cycle burst, representing the steady state excitation^34^ with PNP varied between 0 to 500 kPa. The transducer response was calibrated for each PNP prior to each experiment, by introducing degassed saline into the cellulose fiber, recording the spectra following insonation and subtracting this background signal from the echoes obtained throughout the experiments. Next, MBs were injected into the fiber and their corresponding response was recorded. The same process was repeated with a transmitted frequency of 1 MHz.

### ***In vivo*** transcranial ultrasound parameters

All animal-related work performed by our laboratory was in accordance with the Guide for the Care and Use of Laboratory Animals of the National Institutes of Health and all animal experiments were performed under a protocol approved by the Institutional Animal Care and Use Committee of the University of California, Davis. To determine the stable cavitation region where safe BBB disruption occurs with 250 kHz ultrasound, 7 groups of FVB mice (Charles River) were studied with the following protocols: (1) no treatment control (NTC) (n = 3), (2) ultrasound-only (no MBs) with a PNP of 500 kPa (twice the highest PNP used with MBs + ultrasound) (n = 3), (3) MBs + 75 kPa PNP (n = 3), (4) MBs + 100 kPa PNP (n = 6), (5) MBs + 150 kPa PNP (n = 6), 6) MBs + 190 kPa PNP (n = 3), and (6) MBs + 250 kPa PNP (n = 1). Contrast enhancement observed in the T1-weighted MR images was used to evaluate the BBB disruption. Prior to the experiment, fur around the treated area was shaved and then further removed using depilatory cream. Ultrasound gel was used as coupling agent. Anesthesia was induced with 2% isoflurane in oxygen (2 L/min). The mouse was positioned in the supine position at the focal depth of the transducer (z = 45 mm). Sonication was performed after injection of a 50 µL bolus of 4 × 10^7^ in-house made MBs (average radius of 0.75 µm) into the tail vein of the animal. Sonication included ultrasound bursts of 10 ms, pulse repetition frequency of 0.2 Hz, and a total of 20 bursts.

At this low frequency, and for the thin skull of a mouse (thickness of ~0.4 mm), attenuation of the beam is negligible. To validate this assumption, a piece of *ex vivo* mouse skull was placed in the water tank between the transducer and the needle hydrophone. Pressure measurements with the skull positioned at the same orientation as in the *in vivo* experiments, showed no attenuation compared to water. Hence it can be concluded that the measured pressure behind the skull is attenuated only by the distance of the soft tissue that the beam propagates in (0.5 dB/cm/MHz), and not by the skull.

### Magnetic resonance imaging

At 0 and 3 hours following ultrasound treatment, BBB opening was assessed using a Bruker Biospec 70/30 (7 T) small animal magnetic resonance imaging (MRI) scanner (Bruker BioSpin MRI, Ettlingen, Germany). The MRI system was equipped with a 72 mm internal diameter volume RF coil for signal transmission and a four-channel mouse brain phased array coil for signal reception. Animals were injected with gadoteridol (0.3 mmol/kg) intraperitoneally and imaged axially and coronally with a T1-weighted sequence (RARE: RARE factor = 2, TR = 300 ms, TE = 6 ms, NEX = 6; FOV = 19.2 × 19.2 mm^2^; MTX = 192 × 192, ST/SI = 1 mm/1 mm). Data were acquired and images were reconstructed using ParaVision 5.1 (Bruker BioSpin MRI). Assessment of contrast enhancement due to BBB opening was performed by calculating the contrast enhancement at the treated area relative to a non-treated area.

### Histological evaluation

A subset of mice from each treated group (75, 100 and 150 kPa ultrasound and MBs) were monitored for 48 hours and were then euthanized. All other mice were euthanized at the end of the sonication day. Post euthanasia, the mouse brain was immediately harvested. The brains were maintained in 10% formalin solution for 7 days and underwent coronal sectioning in the area where the BBB was opened, according to the MRI assessment. Hematoxylin and eosin staining was used to investigate these paraffin-embedded, 4 μm thick brain sections.

## Results

### Numerical evaluation results

The Blake threshold predicts the growth of MB oscillations above a pressure threshold for excitation frequencies that are well below the resonance frequency of the MB (f_0_ ≪ f_c_,). From Equation 1, for a clean gas MB, P_B_ is expected to be 175 and 140 kPa for MB radii of 0.75 and 1.5 µm, respectively. Therefore, for the 250 kHz center frequency (over an order of magnitude from MB resonance), MB oscillations were characterized below and above the Blake threshold pressure.

**Figure 2:**
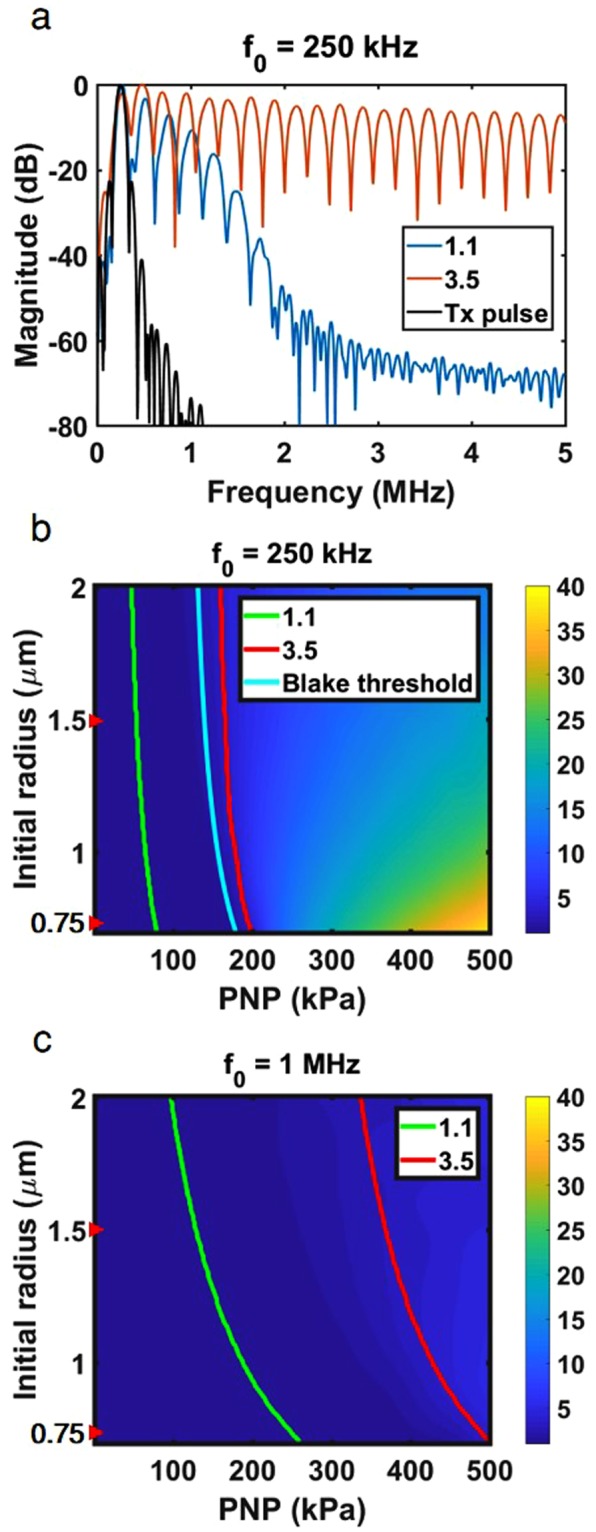
Theoretical predictions using the modified Rayleigh-Plesset Equation of MB oscillation at 250 kHz and 1 MHz with a three-cycle excitation. (**a**) Received spectrum for the transmitted pulse (Tx pulse) and echoes corresponding to 1.1-fold (75 kPa) and 3.5-fold (190 kPa) expansion following 250 kHz transmission, for 0.75 µm radius MBs. Maximal expansion as a function of MB radius and peak negative pressure (PNP) with an expansion of 1.1 and 3.5 fold indicated by the green and red lines, respectively, and the Blake threshold by the blue line (**b**) for a center frequency of 250 kHz. (**c**) for a center frequency of 1 MHz.

A range of PNP from 0 to 500 kPa was evaluated at center frequencies of 250 kHz and 1 MHz. We particularly focused on finding the PNP range that yields expansion ratios between 1.1 and 3.5 as approximate limits of stable and inertial cavitation, as previous literature has reported an upper limit of inertial cavitation between 2.3 and 3.5^13,49^. The RPE predicts that in response to a 75 kPa, 250 kHz pulse, harmonic emissions are evident in the received echo spectrum from a 0.75 µm radius MB resulting in a 1.1-fold expansion ratio. Predicted echo bandwidth increased with an increase in the transmission pressure to 190 kPa and resulting 3.5-fold expansion ratio (Fig. 2a).

The RPE predicts a faster increase in expansion ratio at 250 kHz compared to 1 MHz (Fig. 2b,c). With 250 kHz transmission, MB expansion increases rapidly with a small increase in the transmitted pressure. For smaller MBs (~0.75 μm radius) and the 250 kHz transmission frequency, the MB expansion ratio increases nonlinearly and reaches 35-fold at 500 kPa (Fig. 2b), compared to 3.5-fold at 1 MHz for the same PNP. For larger MBs (~1.5 μm radius), the expansion ratio is smaller and reaches 20-fold and 4.5-fold for 250 kHz and 1 MHz, respectively, at a PNP 500 kPa.

Based on the distance between the green and red lines in Fig. 2b,c, we observe that the stable cavitation pressure range is narrower for the 250 kHz center frequency than for the 1 MHz center frequency (~a factor of 2). The onset of inertial cavitation for 250 kHz occurs at pressures below 200 kPa, and in close proximity to the Blake threshold. For example, for the 250 kHz center frequency, the range of pressures for which the MB expansion ratio remains between 1.1 and 3.5-fold is 75 to 190 kPa for 0.75 µm radius MBs and 55 to 165 kPa for 1.5 µm radius MBs, compared to 245 to 500 kPa and 125 to 400 kPa for the same MB size at 1 MHz.

### Ultra high speed imaging results

**Figure 3:**
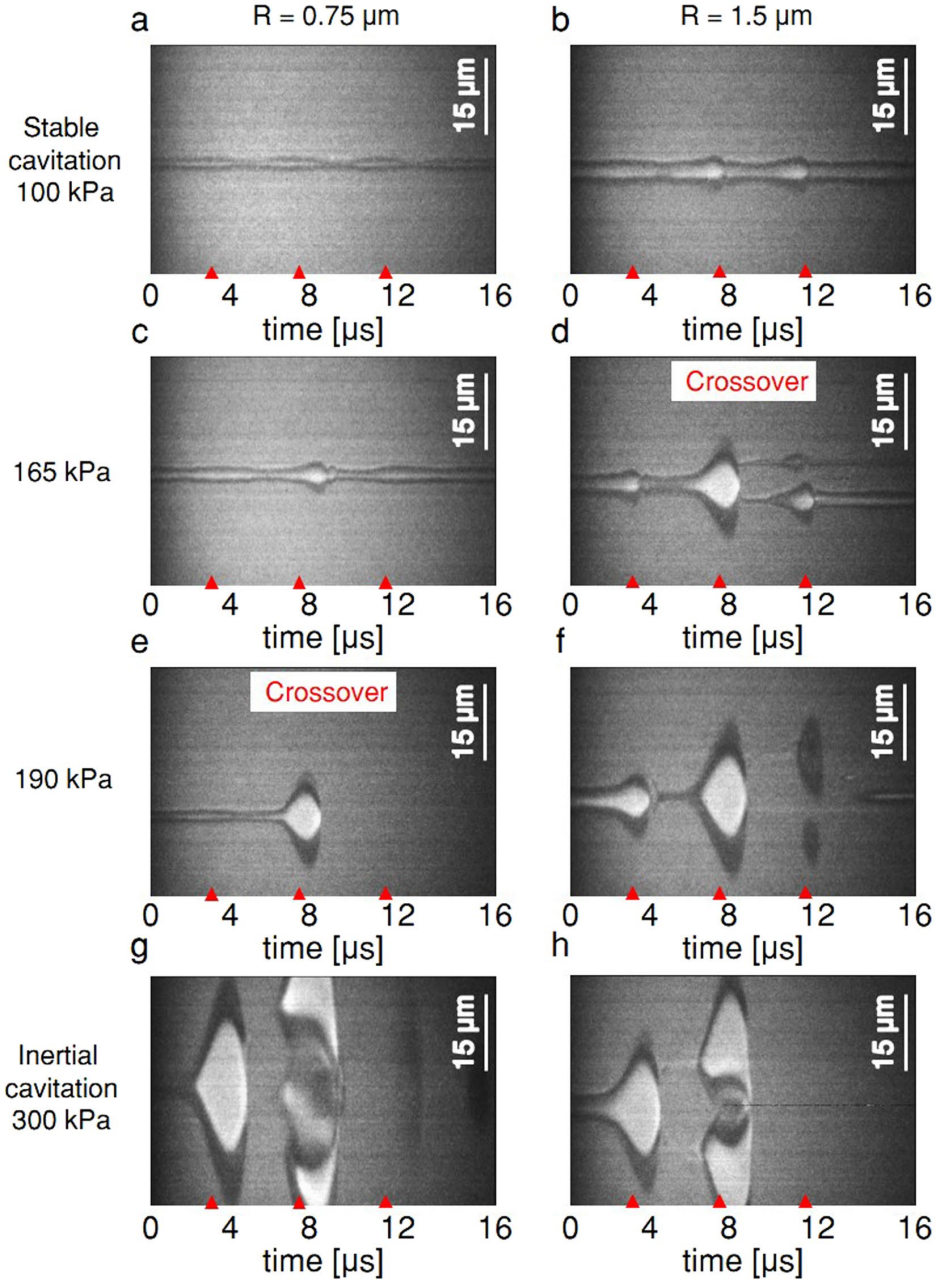
Ultra high-speed camera streak images of oscillating MBs for a transmission center frequency of 250 kHz with radii of 0.75 μm and 1.5 μm at various peak negative pressure (PNP). (**a**,**b**) 100 kPa. (**c**,**d**) 165 kPa. (**e**,**f**) 190 kPa. (**g**,**h**) 300 kPa.

We experimentally validated these predictions by observing single MBs insonified at a center frequency of 250 kHz using an ultra high-speed camera to capture the streak image of the diameter of the MB as a function of time. Images of ~250 single oscillating MBs were captured and processed. Representative streak images at four PNP values are presented in Fig. 3, where the red tick marks indicate the 4 µs period of the three-cycle excitation waveform. At a PNP of 100 kPa, stable cavitation was observed for both 0.75 and 1.5 µm radii (Fig. 3a,b). The crossover from stable to inertial cavitation occurred below a PNP of 165 kPa and 190 kPa for the 1.5 µm and 0.75 µm MBs, respectively. As a result, the smaller 0.75 µm MBs remained intact following insonation at 165 kPa (Fig. 3c), while the larger MB fragmented (Fig. 3d). A pressure of 190 kPa resulted in fragmentation of the 0.75 µm MB, hence at this pressure both MBs were observed to oscillate and fragment (Fig. 3e,f). At 300 kPa, expansion to a diameter greater than 45 µm was followed by fragmentation (Fig. 3g,h).

**Figure 4:**
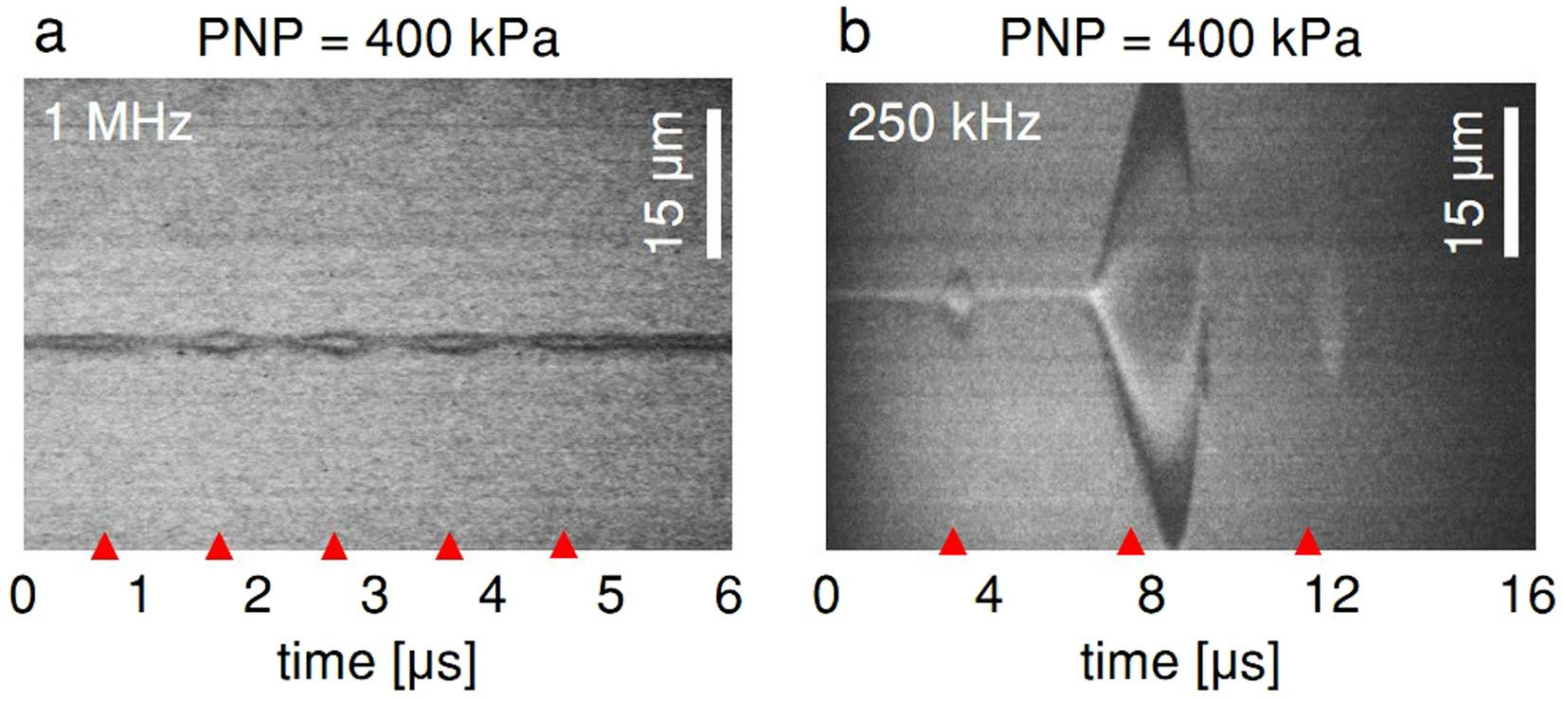
Ultra high-speed camera streak images of oscillating MBs with a resting radius of 1.5 µm for a peak negative pressure (PNP) of 400 kPa at a center frequency of (**a**) 1 MHz. (**b**) 250 kHz.

Increasing the PNP to 400 kPa resulted in a maximal expansion ratio of 1.6 for 1 MHz insonation (Fig. 4a), in agreement with the theoretical predictions. In comparison, for a center frequency of 250 kHz, an expansion ratio of 30 fold was observed with the same PNP (Fig. 4b).

**Figure 5:**
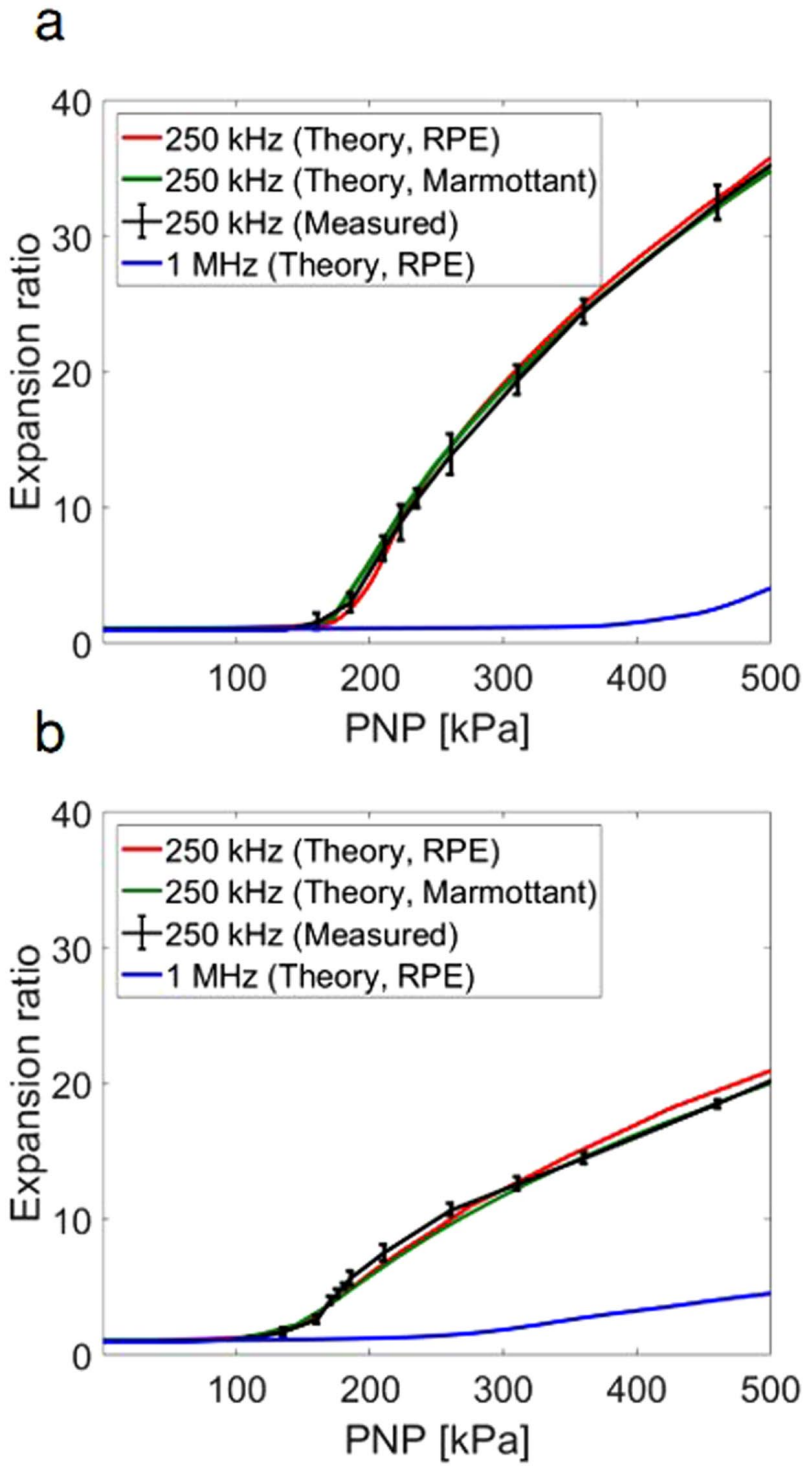
Ultra high-speed camera experimental observations and theoretical predictions for MB expansion ratio using the modified Rayleigh-Plesset Equation (RPE) and the Marmottant model, as a function of peak negative pressure (PNP) for a transmission center frequency of 250 kHz and 1 MHz. (**a**) for MBs with radii of 0.75 μm. (**b**) for MBs with radii of 1.5 μm.

Image post-processing determined the resting MB diameter and expansion ratio for experimental measurements (n = 4 each parameter set). With the 250 kHz center frequency, experimental observations of the expansion ratio as a function of PNP were similar to the RPE and the Marmottant model predictions for MBs with radii of 0.75 μm (Fig. 5a) and 1.5 μm (Fig. 5b). The expansion ratio increases rapidly as a function of pressure and reaches 35 and 17-fold at 500 kPa for the 0.75 and 1.5 μm resting radii, respectively.

### Passive cavitation detection results

**Figure 6:**
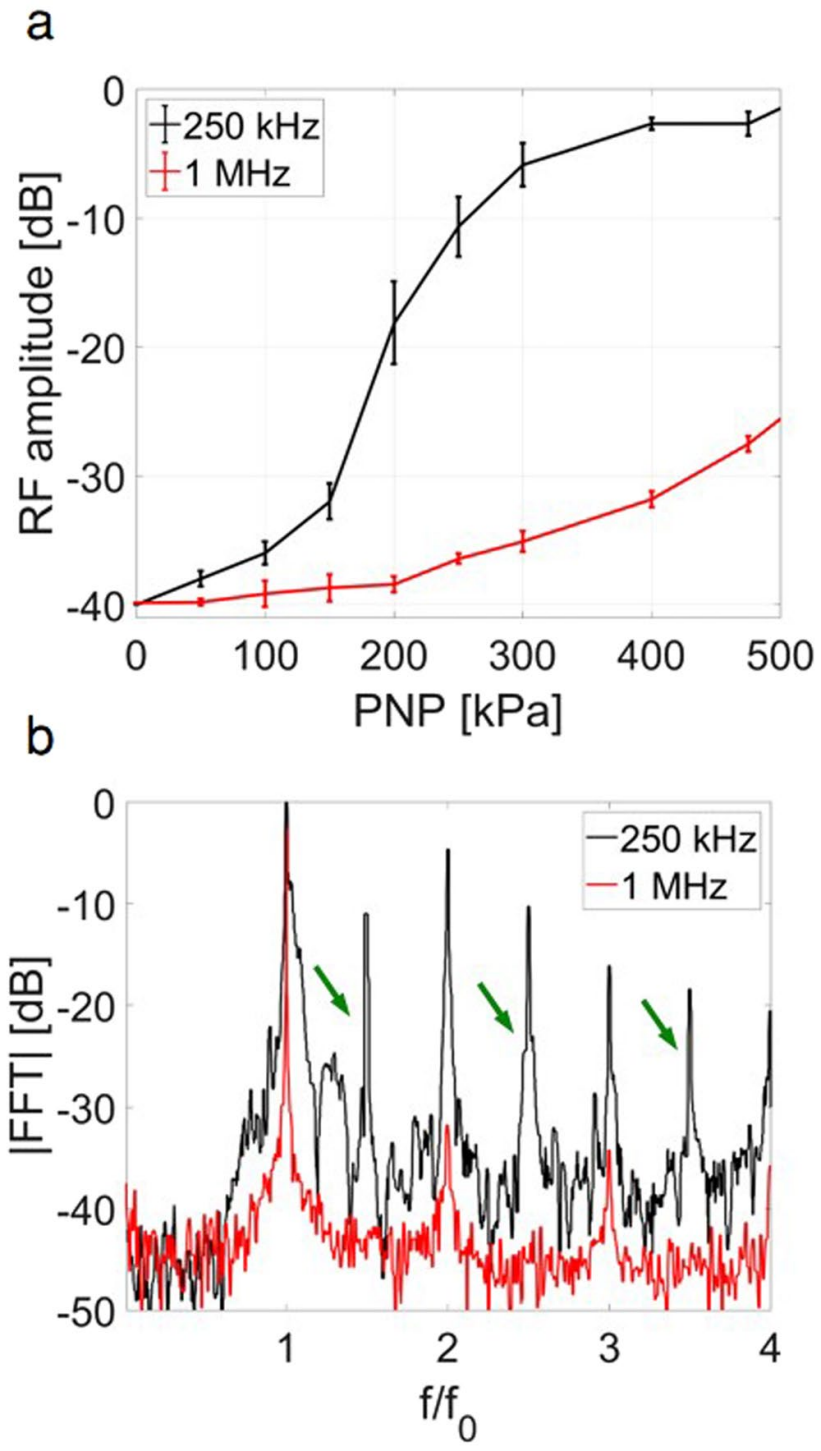
Passive cavitation detection. (**a**) RF amplitude of the second harmonic as a function of the peak negative pressure (PNP). Ultraharmonic components at 250 kHz insonation are indicated by the green arrows (**b**) Magnitude of the received echo spectrum following excitation of the MBs as a function of normalized frequency using 400 kPa and a center frequency of 250 kHz (black), and 1 MHz (red).

The cavitation activity excited by a 250 kHz or 1 MHz burst for each PNP was then estimated using the second harmonic amplitude^52^ recorded passively using a 1-MHz transducer (Fig. 6a). The acoustical results follow the same trend of the theoretical predictions and the optical observations. For a center frequency of 250 kHz (black line, Fig. 6a), the signal increases linearly until ~175 kPa, followed by a non-linear increase in signal that is associated with inertial cavitation. The threshold for a rapid increase in the echo is similar to the optical and acoustical measurements; however, the slope of the increase differs, as expected, since the MB echo varies with the second derivative of the radial fluctuations. For a center frequency of 1 MHz (red line, Fig. 6a), the increase in expansion ratio begins at a higher PNP (400 kPa). The amplitude of the second harmonic at 500 kPa is reduced by 25 dB compared to a frequency of 250 kHz.

The spectral components further indicate inertial cavitation is associated with ultraharmonic components and increased broadband energy. At 400 kPa, the expansion ratio of MBs with the 1 MHz insonation is 1.6 (shown earlier in Fig. 4a). In this stable cavitation range, harmonic emission is expected (red line, Fig. 6b). Alternatively, at 250 kHz, for the same PNP of 400 kPa, the expansion ratio is 30 (shown earlier in Fig. 4b). Thus, strong inertial cavitation, with ultraharmonic amplitude similar to the harmonic components, and wideband signals are evident (black line, Fig. 6b).

### *In vivo* transcranial ultrasound results

**Figure 7:**
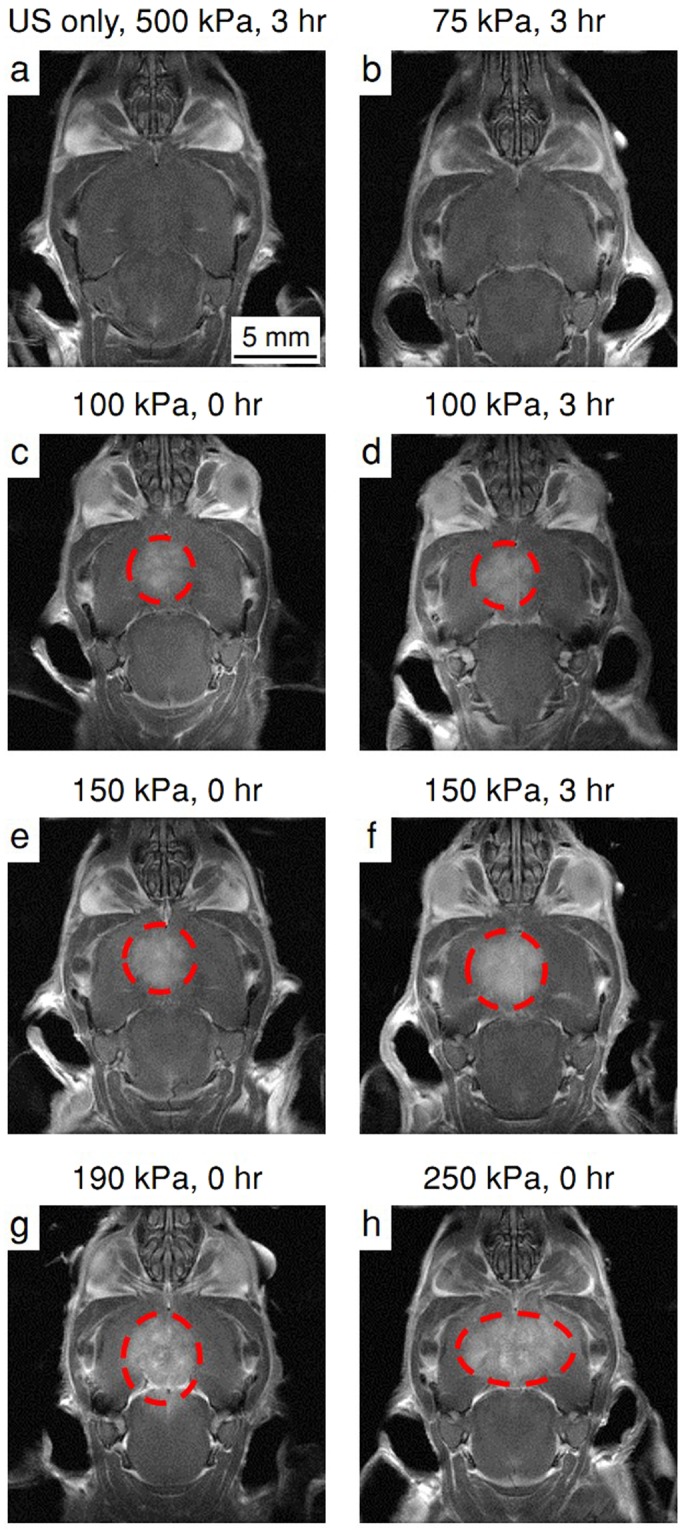
Coronal T1-weighted MR images. The contrast enhancement area is indicated by the red dashed circles. (**a**) US (ultrasound)-only with 500 kPa peak negative pressure (PNP), 3 hours post treatment. (**b**) ultrasound + MBs with 75 kPa PNP, 3 hours post treatment. (**c**,**d**) Ultrasound + MBs with 100 kPa PNP, at 0 and 3 hours post treatment respectively. (**e**,**f**) Ultrasound + MBs with 150 kPa PNP, at 0 and 3 hours post treatment respectively. (**g**) Ultrasound + MBs with 190 kPa PNP, at 0 hours post treatment. (**h**) Ultrasound + MBs with 250 kPa PNP, at 0 hours post treatment.

**Figure 8:**
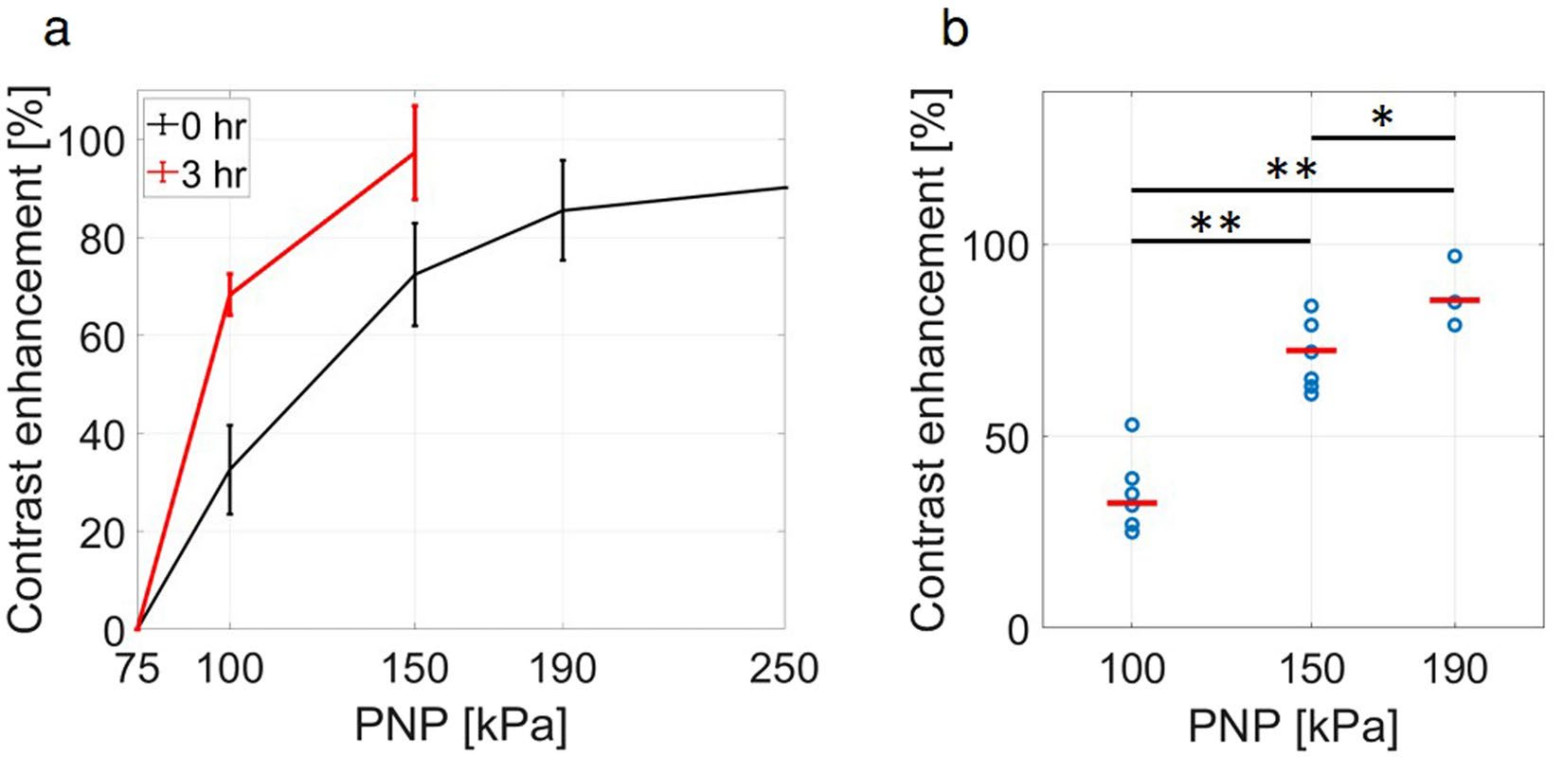
Contrast enhancement observed using magnetic resonance imaging (MRI). (**a**) Contrast enhancement as a function of ultrasound peak negative pressure (PNP) at 0 and 3 hours post treatment. (**b**) Contrast enhancement increases with the PNP of the therapeutic ultrasound pulse at 0 hours post treatment. *Indicates p < 0.05, and **indicates p < 0.01.

To complement the *in vitro* MB characterization, we sought to evaluate the range of pressure resulting in safe and effective BBB opening *in vivo* over a PNP range of 75 to 250 kPa. Contrast enhancement observed in the T1-weighted MR images was used to evaluate the BBB disruption. For the control groups of NTC and ultrasound-only (Fig. 7a), no contrast enhancement was observed. The group treated with 75 kPa PNP ultrasound and MBs (Fig. 7b), showed no contrast enhancement. In all groups treated with ultrasound and MBs, at a PNP of above 75 kPa, successful BBB opening in the sonicated area was observed (Fig. 7c–h). Mice treated with a PNP above 150 kPa (e.g. 190 kPa (Fig. 7g) and 250 kPa (Fig. 7h)) showed signs of acute neurological damage and were euthanized post treatment. For all other mice, contrast enhancement was additionally evaluated 3 hours post treatment. Contrast agent accumulation was not enhanced in the NTC, the ultrasound-only cohorts, and the mice treated with 75 kPa PNP ultrasound and MBs compared to the 0-hour time point. Contrast agent accumulation was enhanced in mice insonified with the 100 and 150 kPa PNPs at 3 hours post treatment compared to the 0-hour time point (Fig. 7d,f compared to Fig. 7c,e and Fig. 8a). Comparing the results of histology and the contrast enhancement, we find that a contrast enhancement of above 80% is associated with acute damage in the corresponding treated mice. Further, we find that there is a significant difference between the groups that were treated with different PNPs (Fig. 8b).

**Figure 9:**
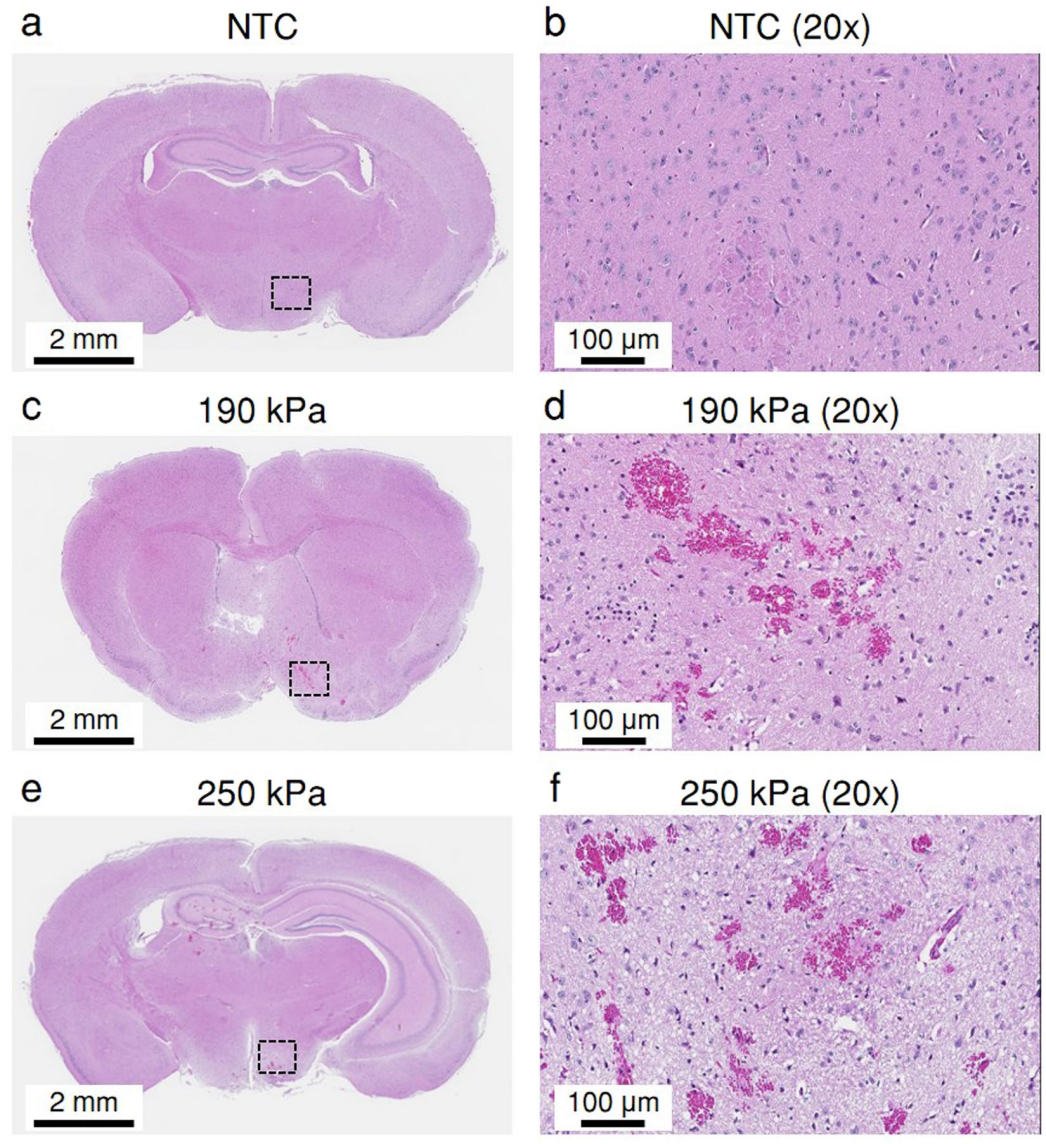
Histological photomicrographs obtained 1 hour post treatment. (**a**) No treatment control (NTC). (**b**) 20x magnification of the dashed black square area in a. (**c**) MBs + 190 kPa peak negative pressure (PNP) ultrasound. (**d**) 20x magnification of the dashed black square area in c. (**e**) MBs + 250 kPa PNP ultrasound. (**f**) 20x magnification of the dashed black square area in e.

No changes were observed on histological evaluation for the NTC (Fig. 9a,b), ultrasound-only, and ultrasound + MBs 75, 100 and 150 kPa cohorts. No changes in five veterinary health measures (activity, weight, food intake, posture and hydration) were observed, and no pathological changes were detected in mice that were monitored for 48 hours post treatment. In the histological images associated with the higher PNPs of 190 (Fig. 9c,d) and 250 kPa (Fig. 9e,f), lesions were evident. Edema and acute micro vessel hemorrhage were observed through the presence of extravascular red blood cells, in addition to perivascular infiltration and vascular congestion.

## Discussion and Conclusions

Opening of the blood brain barrier to deliver therapeutics is an important goal. When commercial MB contrast agents oscillate in stable cavitation, transport to the brain can be safely accomplished. However, large oscillations and transient cavitation can damage delicate brain tissue and must be avoided^18,19^. Currently, an ultrasound center frequency near 250 kHz is proposed for therapeutic clinical human studies to open the blood brain barrier, rather than the intensively-studied MHz insonation frequency range^18^. The use of such a low ultrasound transmission center frequency is desirable to enhance penetration depth, enlarge the focal zone to reduce treatment time for larger clinically-important targets, minimize distortion and attenuation, and to facilitate beam steering throughout the brain using typical undersampled 2D therapeutic transducer arrays. This 250 kHz frequency is an order of magnitude below the resonance frequency of ultrasound contrast agents. While many previous studies have focused on resonant MB oscillation and assumed that oscillation is maximized at resonance, here we demonstrated that high amplitude oscillations can occur far from resonance, even for encapsulated MBs.

Our results indicate that MB expansion is enhanced for 250 kHz transmission as compared with the 1 MHz center frequency. In fact, predicted and measured expansion ratios reached *30-fold* with 250 kHz at a PNP of 400 kPa, as compared to a measured expansion ratio of *1.6-fold* for 1 MHz transmission at a similar PNP. Both *in vitro* and *in vivo*, we found that the oscillation of lipid-shelled MBs was similar to that of clean gas bubbles at this low frequency, and the effect of the MB shell was negligible due to the exposed gas/water interface resulting from the large expansion. The Marmottant model accounts for the surface tension modifications of the MB shell and its rupture for large oscillations. Under these assumptions, the MB behaves as a clean gas MB once the expansion extends beyond the rupture radius^53^—an inherent result of the Marmottant model for large expansion ratios which allows this model to predict the expansion ratio at 250 kHz. Similarly, numerical results obtained with the RPE, parameterized for a clean gas MB, were also consistent with the experimental observations.

The *in vitro* experiments were performed with MBs housed within a 200 µm cellulose tube. It was previously reported that when the distance between a MB and the tube wall is larger than 25 times the MB resting diameter, the MB behaves as if it is in an infinite fluid^54^. Here, since the MB diameters were less than 3 µm, the tube diameter was 200 µm, and the MBs imaged with the ultra high-speed camera were located at the center of the tube, their distance from the tube wall fulfills the above condition. Additionally, regardless of the vessel rigidity index, the natural frequency of a 3 µm MB oscillating in a 200 µm vessel is not significantly altered as described in Figs 3 and 5 in^35^. The assumption that the oscillation is similar to that of MBs within an infinite fluid is further validated by the similarity between the theoretical predictions of MBs oscillations assuming infinite space and the experimental results.

The Blake threshold has previously been applied to predict high amplitude oscillations of clean gas bubbles in the quasi static state (i.e. when they are insonified well below their resonance frequency) and predicts infinite growth beyond a static tension threshold. Since the resonance frequency for such clean gas MBs is 2.5 and 6 MHz for MB radii of 0.75 and 1.5 µm, the Blake threshold can be applied for 250 kHz insonation but not for 1 MHz insonation. The resulting Blake pressure threshold for low frequency insonation of MB radii of 0.75 and 1.5 µm is 175 and 140 kPa, respectively. These pressures are in agreement with the onset of inertial cavitation for encapsulated MBs as detected with the ultra high-speed camera imaging. We found that the Blake threshold can be accurately applied to predict the expansion of lipid-shelled MBs in the 250 kHz frequency range.

We emphasize that our study is limited to lipid-shelled MBs. However, the pressure threshold for the onset of inertial cavitation with shell materials such as albumin or polymer is expected to be different. Further study is needed to quantify the 250 kHz PNP range transitioning from stable cavitation to inertial cavitation for such materials.

### ***In vivo*** results validated enhanced BBB opening with parameters similar to ***in vitro*** work

*In vivo* transcranial ultrasound at a center frequency of 250 kHz combined with MB injection in mice showed localized uptake of an MR contrast agent and confirmed BBB disruption at a PNP of 100–250 kPa. However, at PNPs above 150 kPa, inertial cavitation-induced mechanical lesions were evident. This indicates that the *in vivo* transition to inertial cavitation occurs near a PNP of 190 kPa and follows the same trend as MB oscillations in 200-µm cellulose fibers. Our previous work showed that (1) smaller vessels have a constraining effect on MB oscillations^45^, and also that (2) MB-mediated permeability changes can occur in vessels as large as 50 µm where the largest oscillations will occur^55^. Given that the microvasculature is composed of vessels with a range of diameters, we chose to study oscillation in larger vessels recognizing that the oscillation in the tubing used here will be similar to that in 40 µm or larger vessels, that this will be the largest oscillation that will occur, and that vascular changes do occur in vessels in this size range.

Results from our study confirm that the contrast enhancement estimation may hold a predictive value in assessing the safety of the treatment. When the ultrasound PNP is such that the MBs are in inertial cavitation, the treatment effect moves beyond a transient change in permeability and can potentially induce damage to brain tissue. The relationship between contrast enhancement and the duration of BBB permeability enhancement has been previously reported^56^, as well as the relationship between contrast enhancement, the magnitude of inflammatory response^57^ and changes in gene expression^58^. In this study, contrast enhancement below 80% did not result in a detectable injury. This correlation may help guide the safety standards for our particular experimental setup. However, the exact relationship between contrast enhancement and the safety of the treatment depends on the specific experimental setup, the ultrasound parameters, the species, the region of brain sonicated, the concentration of MRI contrast agent in circulation, the type of MRI contrast agent administered, etc. Therefore, the exact relationship should be established per experimental configuration.

### Study considerations

The numerical evaluations assume that the MBs remain spherical during stable and inertial cavitation. Indeed, during inertial cavitation, MBs can fragment asymmetrically. Here, only streak images that exhibited radial symmetry oscillations were selected and analyzed. The ultra high-speed camera captures images with a temporal resolution of 28 ns, and an acquisition period of 16 µs (corresponding to 4 cycles of the 250 kHz transducer). Hence, we chose a three-cycle excitation for acquisitions with the ultra-high speed camera in order to visualize the entire oscillation. Since the maximal expansion ratio for inertial cavitation is achieved during the second cycle, the acquisition of three cycles was sufficient for the analysis of the expansion ratio that was performed here; Further, the maximal expansion analysis is also valid for longer transmissions that are used for therapeutic applications. Therapeutic ultrasound typically uses long waveforms, therefore for the cavitation detection and the *in vivo* study, we used 100-cycle bursts. The steady-state response that was characterized using passive cavitation detection and followed a similar trend as the results obtained with the ultra high-speed imaging.

In summary, the development of safe and successful protocols for therapeutic delivery to the brain, utilizing 250 kHz or a similar center frequency requires consideration of the narrow pressure window between stable and inertial cavitation. As a result, passive monitoring of the MB cavitation activity during an ultrasound treatment is particularly important in this low frequency range.

## Data Availability

The datasets generated during and/or analyzed during the current study are available from the corresponding author on reasonable request.
